# Ship-Fire Net: An Improved YOLOv8 Algorithm for Ship Fire Detection

**DOI:** 10.3390/s24030727

**Published:** 2024-01-23

**Authors:** Ziyang Zhang, Lingye Tan, Robert Lee Kong Tiong

**Affiliations:** School of Civil and Environmental Engineering, Nanyang Technological University, Singapore 639798, Singapore; ziyang004@e.ntu.edu.sg (Z.Z.); clktiong@ntu.edu.sg (R.L.K.T.)

**Keywords:** ship-fire detection, YOLOv8n, deep learning, GhostnetV2, ODConv, SCConv

## Abstract

Ship fire may result in significant damage to its structure and large economic loss. Hence, the prompt identification of fires is essential in order to provide prompt reactions and effective mitigation strategies. However, conventional detection systems exhibit limited efficacy and accuracy in detecting targets, which has been mostly attributed to limitations imposed by distance constraints and the motion of ships. Although the development of deep learning algorithms provides a potential solution, the computational complexity of ship fire detection algorithm pose significant challenges. To solve this, this paper proposes a lightweight ship fire detection algorithm based on YOLOv8n. Initially, a dataset, including more than 4000 unduplicated images and their labels, is established before training. In order to ensure the performance of algorithms, both fire inside ship rooms and also fire on board are considered. Then after tests, YOLOv8n is selected as the model with the best performance and fastest speed from among several advanced object detection algorithms. GhostnetV2-C2F is then inserted in the backbone of the algorithm for long-range attention with inexpensive operation. In addition, spatial and channel reconstruction convolution (SCConv) is used to reduce redundant features with significantly lower complexity and computational costs for real-time ship fire detection. For the neck part, omni-dimensional dynamic convolution is used for the multi-dimensional attention mechanism, which also lowers the parameters. After these improvements, a lighter and more accurate YOLOv8n algorithm, called Ship-Fire Net, was proposed. The proposed method exceeds 0.93, both in precision and recall for fire and smoke detection in ships. In addition, the mAP@0.5 reaches about 0.9. Despite the improvement in accuracy, Ship-Fire Net also has fewer parameters and lower FLOPs compared to the original, which accelerates its detection speed. The FPS of Ship-Fire Net also reaches 286, which is helpful for real-time ship fire monitoring.

## 1. Introduction

Oceans encompass approximately 71% of the Earth’s surface, giving rise to extensive water bodies that serve as natural channels. Since the 15th century, the rapid growth of maritime transportation has facilitated intercontinental mobility for humans, leading to extensive exchanges of people and goods that have had profound impacts on both social and natural environments. Historically, the majority of international trade, approximately 80–90%, has been facilitated through maritime transportation. Ships play a pivotal role in the domain of maritime transportation, serving as crucial facilitators for the transportation of commodities, people, and assets over the vast expanse of the Earth’s oceans, seas, rivers, and other aquatic passages. Although shipping is often regarded as an efficient mode of transportation, it poses significant risks to the safety and well-being of all ships aboard. A common occurrence in the shipping industry is the potential hazard of fire, which poses a significant risk. A ship fire disaster is a highly consequential incident that poses a significant risk to the safety of maritime transportation, and may result in the loss of the ship, damage to cargo, environmental contamination, human injuries, and even fatalities [1]. According to the Safety and Shipping Review 2023 conducted by Allianz [2], the world’s most well-known and largest insurer and financial services group, fire was as the second most prevalent factor contributing to the loss of shipping vessels in the previous year. There are about a total of 250,000 claims within the maritime insurance market, and it has been shown that fire was the costliest source of loss, representing 18% of the total value of all claims that were examined. However, with the development of technology and training for safety awareness and the professional skills crew members have shown on encountering risks, there has been a significant decline in shipping loss in the previous decade (eight vessels in 2022 compared with over 100 in 2013). However, the number of fire accidents in ships does not follow this trend. In 2022, there were a total of 209 recorded fire occurrences, which is the highest number in a decade. In the previous five years, a total of 64 ships were lost due to fires. There have been large numbers of devastating ship fire accidents in recent years. For instance, in January 2022, one ship carrying more than 4000 cars, including Porsche, Audi, Bentley, and Lamborghini models, caught fire in the mid-Atlantic. The projected economic impact of this unfortunate event was approximately USD 438 million worth of goods. In addition to economic losses, fire-in-fire may also threaten human lives and safety. According to CNN, two firemen perished while engaged in combat against conflagration, and a minimum of at least six other individuals sustained injuries. Almost 1200 vehicles and 157 containers burst onto the ground, resulting in significant economic losses. Moreover, ship fires can have a significant environmental impact, as the combustion of fuel, cargo, and other ship components results in the emission of pollutants into both atmospheric and aquatic environments. In addition, ships can transfer hazardous and toxic chemicals, resulting in severe damage to the environment. For instance, on 20 May 2021, a cargo ship carrying tons of hazardous and toxic chemicals caught fire near Colombo’s commercial harbor, resulting in hazardous poisonous substances discharged into the ocean. These examples prove that fire accidents in ships may result in economic loss and threaten health and environmental hazards. Early detection of fire and smoke can both give time for crew members and passengers to evacuate or put out the fire to lower the loss. A 10-min delay in extinguishing a fire in the engine room might potentially incur a financial loss of USD 200,000; however, a 20-min delay could result in a tenfold increase in the loss [3].

Currently, there are three fundamental approaches for fire identification: human inspection, sensor-based detection, and image processing technology. Human inspectors who have received formal training possess specialized knowledge and practical skills that are useful for carrying out their duties. They can discern tiny indications of potential fire risks and demonstrate proficiency in evaluating intricate circumstances. However, there is a remarkable shortage of human inspections. Assessments conducted by human inspectors may be influenced by subjective factors, resulting in the uneven rating of fire safety measures. The accuracy and dependability of inspections may also be influenced by several factors, including human prejudice and judgment. Additionally, inspectors may experience weariness while engaging in extended and repeated inspection activities, thereby increasing the probability they will overlook or fail to identify crucial safety concerns. Manual inspections are also susceptible to the inherent dangers of human error. These shortcomings result in high economic costs.

With regard to sensor-based detection, sensor-based systems can identify fires during their first phases, often before the emergence of visible flames or smoke. The provision of an early warning system enables expedited reaction durations and heightened fire-suppression probabilities before fire propagation. In addition, sophisticated sensor technologies have been developed to reduce the occurrence of false alarms by effectively differentiating between genuine fire incidents and other extraneous elements, such as cooking smoke or dust present in the environment. Although sensor-based detection has these advantages, it also has some shortcomings: sensors have restricted coverage ranges, resulting in diminished efficacy in expansive, unenclosed environments or locales characterized by blocked sightlines. In order to provide sufficient coverage over large regions, it may be necessary to use many sensors, resulting in higher costs. Moreover, regular maintenance and calibration of sensors are necessary to guarantee their accuracy and dependability. Failure to prioritize maintenance may result in inaccurate alerts or, even more concerning, a failure to identify critical events. Compared with the first two methods of fire detection, image processing has the advantages of cost-effectiveness and efficiency, and its accuracy has shown consistent improvement with the development of improved processing power and the availability of specialized hardware, such as GPUs. Consequently, some researchers have used image-processing methodologies to identify and classify flames. In being applied to fire detection, conventional image processing methods often rely on manually selected characteristics, such as color [4,5], texture [6,7], and geometric features [8,9], to effectively partition fire instances. These segmented fires are then subjected to classification and matching procedures that are facilitated by machine learning algorithms, such as support vector machines (SVMs) [10], random forests [11], and K-means [12], to accomplish the task of fire detection. Nevertheless, standard image-processing approaches are inadequate for meeting the requirements of model generalization capability and durability in actual engineering situations via manually created feature extraction, which is mostly due to the complex nature of the fire environment.

Compared to traditional machine learning, where feature extraction is performed by humans, deep learning models can autonomously acquire pertinent characteristics from unprocessed inputs. The use of deep learning obviates the need for human feature engineering, enabling the model to autonomously uncover complicated patterns and representations. In addition, deep learning models, and artificial neural networks in particular, use non-linear activation functions inside their layers. The presence of nonlinearity enables models to effectively capture complex connections within the data, which is a task that linear models frequently used in conventional machine learning sometimes struggle with. Fire detection systems that use deep learning for fire identification are more effective than standard image-processing-based approaches. As a result, there is growing interest in fire detection systems that employ deep learning techniques instead of relying on feature descriptions [13,14].

However, the majority of deep-learning video fire detection systems available on the market require the use of high-performance central processing units (CPUs) and graphics processing units (GPUs) to enhance computational efficiency. Additionally, several embedded platforms are required for picture recognition from cloud-based sources, which may be attributed to the prioritization of real-time recognition in target identification and the substantial computational requirements associated with deep learning. The detection targets of most detection algorithms are normal or forest fires, and not ship fires, which have unique characteristics, when compared to other fires. In normal forest fires, the background is always land or forest, whereas in the case of ship fires, it is always water or the ocean. Different backgrounds may result in the former fire detection algorithm losing its accuracy, because the features of fire with a land background are significantly different from those with a sea background. Although a large number of algorithms use computer vision or deep learning for fire detection [15,16,17,18], most are aimed at fire or smoke detection for forests, rather than ships. The ship fire possesses a different background compared with fire in forests, and this may result in bad performance when using forest fire detection algorithms to detect ship fire. In addition, algorithms for forest fire detection are not sufficiently lightweight to be arranged on ships. And there are only a limited number of deep algorithms that can be used to detect ship fire [19,20,21,22], and all of them possess disadvantages. Park et al. [19] use yolo to detect engine room fires in ships with a high degree of accuracy. However, their detection targets are only fires in engine rooms, meaning that fires on board or in the living rooms are not considered. Xu et al. [20] design a multi-layer convolutional neural network to classify the fire hazard levels in ship cabins. However, all the data are not real data and are simulated by software, which may account for the algorithm not being applicable in the real world. Wu et al. [21] propose an improved YOLOv4-tiny algorithm for ship fire detection. Although the precision and recall increase after modification, both detection time and FPS decrease. Avazov et al. propose an improved YOLOv7 algorithm for ship fire detection. Although the precision and recall improve after modification, the proposed dataset for training may have some shortcomings. About 2400 images are cut from videos, which account for more than half of the dataset: the video frames also have a similar background, which may result in the usage of the trained algorithm being limited. Above all, the existing algorithm has some disadvantages in detecting fire, including the fact they can only detect fire, and not smoke which is an important derivative of fire that can also be detected. In this study, we propose an improved YOLOv8 algorithm that considers both fire and smoke, and which provides a better performance (in terms of both detection speed and accuracy), when compared with other advanced algorithms after experiments. In addition, other improvements are also made to Ship-Fire Net to make it more accurate and faster. The specific work follows:

1. We created a dataset for ship fire detection that contained images of fires in both rooms and boards. After excluding similar images, we created labels manually to ensure the quality of fire and smoke.
2. We broadened the backbone network of YOLOv8n by adding a GhostnetV2-C2F module for long-range attention with an inexpensive operation.
3. We used spatial and channel reconstruction convolution (SCConv) to reduce redundant features with significantly lower complexity and computational costs for real-time ship fire detection.
4. We used omni-dimensional dynamic convolution is the multi-dimensional attention mechanism; on the one hand, this lowers the parameters, and on the other, it improves the models’ capacity to comprehend contextual information and then improve precision.
5. We proposed a lighter and more accurate YOLOv8n algorithm, and named it “Ship-Fire net”. Compared to the original YOLOv8n, it has fewer parameters, fewer FLOPs, and higher FPS when its accuracy is increased.
6. Ship fire nets can also detect smoke, which other ship fire-detection algorithms ignore.

The remainder of the paper is structured as follows: Section 2 discusses the present development status of fire detection technologies and target detection techniques in the areas of machine learning and deep learning. Section 3 presents the improved lightweight convolutional neural method for ship fire detection (“Ship-Fire Net”) in detail. Section 4 illustrates the details of how the datasets were built, and also outlines the establishment of the experimental environment settings and the evaluation index. Section 5 discusses the selection of the original algorithms and ablation tests, and also offers a comparison that considers ship fire nets against ship fire detection algorithms proposed by other researchers. Finally, Section 6 concludes the paper, discusses its limitations and outlines future research directions.

## 2. Literature Review

There are two primary classifications of fire detection strategies for visual recognition: conventional detection approaches that rely on picture characteristics, and detection methods based on deep learning principles. Previous studies in the field of visual identification have primarily used feature extraction techniques, including flame-specific chromatograms, forms, textures, and flame motion. A significant issue associated with these conventional approaches is the intricate nature of the manual feature extraction process. Deep learning-based detection approaches can automatically extract intricate picture details and features, thus efficiently addressing the issues of duplication and interference associated with human image feature extraction. Hence, recent scholarly investigations have primarily focused on the utilization of deep-learning techniques for fire detection, and their outcomes have consistently shown enhanced precision rates and reduced false alarm rates. Fire-detection methods based on color features have also been widely studied.

### 2.1. Machine Learning

In previous research, scientists have used color, texture, flame shape, and flame motion features to identify or detect features. Celik et al. used the [5] $YC_{b}C_{r}$ color rather than RGB to separate luminance from chrominance, and found that $YC_{b}C_{r}$ was more effective. Khalil et al. [23] proposed a novel fire-detection method based on a combination of RGB and CIL L∗a∗b color models. Poobalan et al. [24] used RGB and HSL filters to detect the color of a fire with bag-of-features (BoF), in order to ascertain the classification and calculation of the rate. Vipin [25] presented a new algorithm that uses the RGB and $YC_{b}C_{r}$ color space and tests two sets of images. The Lucas–Kanade optical flow algorithm was proposed to calculate the optical flow of candidate fire and smoke [26], and the determination of potential smoke areas was achieved by using a background estimate and a color-based decision procedure.

Flame-texture feature extraction is often used in the detection and identification of fires, including by Cui et al. [27], who presented a method for analyzing the texture of fire smoke by combining two innovative texture analysis tools: wavelet analysis and gray-level co-occurrence matrices (GLCM). Yu et al. [28] proposed a method for real-time fire smoke detection based on the GLCM, which featured a neural network that could be used to classify smoke texture features from non-smoke texture features. Chino et al. [29] provided an innovative approach for detecting fires in static images, and introduced an approach that used color feature classification in conjunction with texture classification in superpixel areas. Ye et al. [30] proposed a novel dynamic texture descriptor by putting forward a surface transform and hidden Markov tree (HTM) model.

In addition to the extant research that has examined color and texture characteristics, fire detection algorithms that rely on flame shape features and flame motion data have also been used. Foggia et al. [7] applied complementary information based on color, shape variation, and motion analysis, and then combined it with a multi-expert system. Subsequently, a novel descriptor based on the bag-of-words approach was proposed to represent motion. Chi et al. [31] built fire detection systems based on chromatic, dynamic, textural, and contour features by using a novel algorithm to extract moving regions and analyze the frequency of flickers.

As described above, many techniques have been proposed for extracting flame characteristics that have significantly contributed to the progress of visual fire detection and improved its accuracy. However, the detection accuracy has been negatively affected by the human extraction of features under fire conditions, because of their inherent complexity.

### 2.2. Deep Learning

In recent years, the advent of powerful GPUs and TPUs that can handle the intense computational demands of deep learning algorithms has significantly accelerated the development of deep learning. Such developments have also accelerated research into the use of deep learning algorithms for fire detection or motivation. In general, deep learning algorithms can be divided into two categories: one-stage detectors, such as the single-shot multibox detector (SSD) [32], including You Only Looking Once (YOLO) [33], and RetinaNet [34]; and two-stage detectors, such as R-CNN [35], Fast R-CNN [36], Faster R-CNN [37], and Mask R-CNN [38]. The two-stage algorithms operate in two steps: first, regions of interest (ROIs) where objects might be located are proposed, before the bounding boxes of regions are classified, refined and widely used for fire detection. Zhang et al. [39] employed a Faster R-CNN to detect wildland forest fires, and, in seekingto address the scarcity of training data (whose deficiency hampers the development of robust models), generated synthetic smoke pictures, in a process that involves the incorporation of (authentic or simulated) smoke into the forest backdrop. Chaoxia et al. [40] presented an improved and Faster R-CNN method to detect flames, which included a color-guided anchoring strategy and embedded global information-guided flame detection; and Barmpoutis et al. [41] presented a method that combined a Faster R-CNN network with linear dynamic systems (LDS) to locate fire regions.

In addition to two-stage algorithms, one-stage algorithms are also widely used in fire detection, and this is because of their elegant pose structure and faster detection speed. For example, Li et al. [42] tested the fire detection performance of advanced object detection CNN models (Faster-RCNN, R–FCN, SSD, and YOLO v3), and found that YOLO v3 had the highest FPS and precision. Zhao et al. [15] proposed an improved YOLO algorithm, Fire-YOLO, that could be used to detect fires, and particularly small targets, in fire scenarios; Dou et al. [43] used YOLOv5s, in cooperation with a convolutional block attention module (CBAM), along with MobileNetV3, ShuffleNetV2, and GhostNet, to achieve the goal of a lightweight algorithm; and Talaat presented the smart fire detection system (SFDS), which uses the improved YOLOv8 to monitor fires in smart cities.

The research mentioned above was not specifically aimed at ship fires, and mostly concentrated on forest fires or fires in cities. Few studies have focused on ship fires. Kim et al. [44] proposed an innovative method that integrates composite channel data, namely, RGB and IR channels, to enhance the effectiveness of fire detection in image-based systems by using convolutional neural networks (CNNs); Park et al. [19] used YOLO to detect fires in the engine room of a ship. Xu et al. [20] proposed an evaluation model based on a CNN that could determine the hazard levels of different cabins in a real-time fire; Wu et al. [21] proposed a modified YOLOv4-tiny algorithm for detecting ship fires; Avazov et al. [22] used YOLOv7 with an improved E-ELAN (extended efficient layer aggregation network) for fire detection and monitoring; and Zhu et al. [45] improved the YOLOv7-tiny model to enhance fire detection performance in a ship engine room. However, these studies have several limitations, which are shown in Table 1. In this study, we created better datasets and used a lighter algorithm (Ship-Fire Net) than the one applied in the other studies. FPS means frames per second, which is always regarded as an important measure for objection detection. The fire spreads fast in ships and needs to be monitored in real-time, and high FPS ensures that a timely response is forthcoming. In addition, a high frame rate per second (FPS) in surveillance and monitoring systems facilitates the real-time processing of video feeds, allowing prompt identification of fire or smoke as they occur. In our research, we have managed to build a lighter weight algorithm and achieve a higher FPS than preceding studies, and we are confident this will contribute to timely fire detection that will reduce response time when ship fires occur.

## 3. Materials and Methods

### 3.1. Model Structure of the YOLOv8n Network

**Figure 1:**
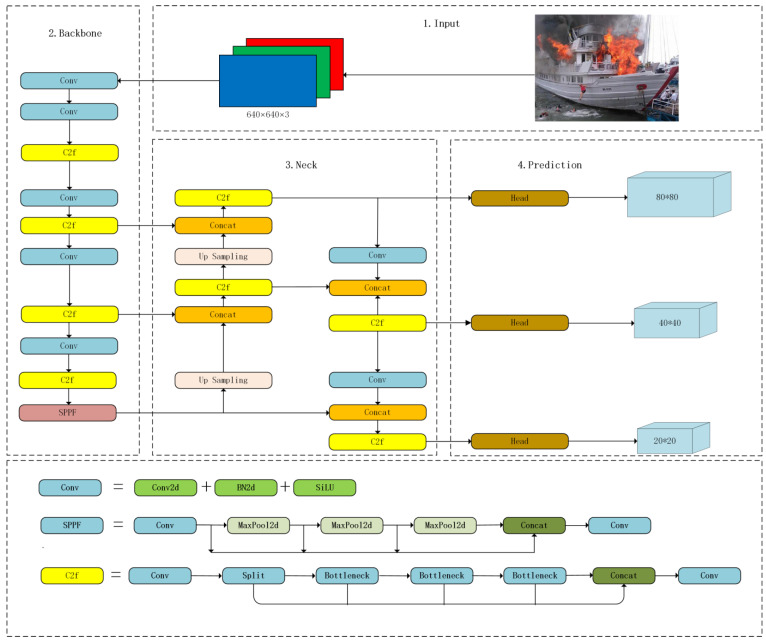
Structure of the YOLOv8n network.

The YOLOv8 model is a sophisticated iteration that integrates the fundamental design ideas of YOLOv5 and YOLOv7 ELAN, its predecessorsYOLOv5 and YOLOv7 ELAN, as shown in Figure 1. The proposed model maintains the essential structure of YOLOv5 while integrating new features and improvements to boost its effectiveness and adaptability. The YOLOv8 model integrates innovative elements, including a redesigned backbone network architecture, an anchor-free detection head, and a revolutionary loss function. The product line offers a diverse range of models of different sizes, including N/S/M/L/X scales that were calibrated by using scaling factors.

The head of YOLOv8 underwent significant modifications, and a decoupled head structure was used to separate the classification and detection heads, before the detection head was converted from an anchor-based to an anchor-free approach. The Loss computation in this study utilized the task-aligned-assignment method to allocate positive samples and distribute focal losses. In addition, a data enhancement component was used by employing the mosaic enhancement strategy during the last ten epochs of the YOLOX model. This methodology has been shown to substantially improve the accuracy of the model. In summary, YOLOv8 is an advanced model that improves the achievements of its predecessor (YOLO), and achieves this by introducing innovative features and enhancements. The product line offers various models of different sizes and incorporates various design adjustments to improve performance and flexibility.

### 3.2. Using GhostNetv2 for Cheap Operation with Long-Range Attention

**Figure 2:**
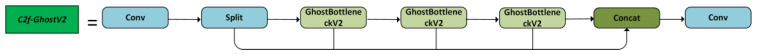
The architecture of the C2F-GhostNetV2 block.

In YOLOv8, conventional convolution and C2F modules were employed to effectively perform feature extraction and image downsampling, resulting in superior quality outcomes. Nevertheless, the inclusion of an upsampling procedure in the neck component and the utilization of the Bi-PAN–FPN resulted in a notable augmentation of both the model parameters and its overall complexity. To reduce the number of parameters and complexity of the backbone, this study incorporates the Ghostblock and double-fully-connected DFC attention idea from Ghostnetv2 [46] into the backbone and uses this structure to replace some C2f modules, in order to achieve cheap operation with long-range attention. GhostNetV2 [29] introduced a hardware-friendly attention mechanism known as DFC attention. This mechanism aims to enhance the long-distance dependence of feature maps by incorporating them after regular convolution and cheapening operations. The goal is to increase the expressiveness and diversity of the feature maps, thereby enhancing the detection performance of the model. The lightweight feature extraction structure C2F-GhostNetV2 was designed by using the benefits of the GhostNetV2Bottleneck, as shown in Figure 2.

**Figure 3:**
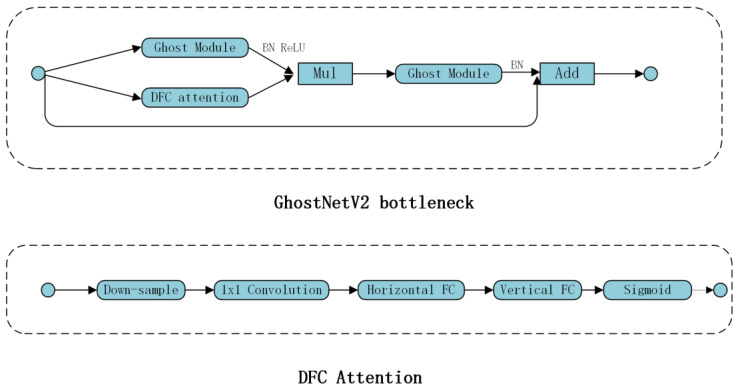
The architecture of the GhostNetV2 bottleneck and DFC attention.

The GhostNetV2Bottleneck is a type of inverse residual bottleneck comprising two ghost modules, as shown in Figure 3. The primary ghost module augments the number of feature channels, whereas the secondary ghost module decreases the channel count to provide output features. The present strategy successfully mitigates the issue of excessive interdependence between model expressiveness and capacity [30], thereby addressing the problem of overfitting during training and inadequate generalization ability during testing. The attention branch of the DFC model is designed to capture the long-range correlation between pixels located at distinct spatial locations. The use of the DFC attention branch in conjunction with the first ghost module augmented the feature characteristics. Subsequently, the improved features are received by the second host module, which generates the output features, thereby improving the performance of the model.

### 3.3. Using SCConv for Feature Redundancy Reduction

**Figure 4:**
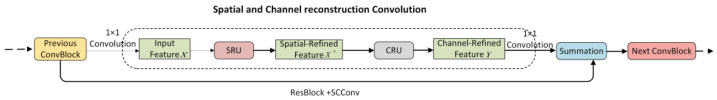
The architecture of SCConv integrated with a SRU and a CRU.

CNNs have been widely used in various computer vision tasks because they can obtain representative features [24]. Nevertheless, achieving such success is strongly dependent on the availability of extensive computational and storage capabilities, which presents significant hurdles to the efficient deployment of these technologies in contexts with limited resources. To address these issues, researchers have investigated many approaches, such as model compression methodologies and network designs, with the aim of enhancing network efficiency. The former includes network pruning, weight quantization, low-rank factorization, and knowledge distillation. While such strategies are useful for compressing the model size, they do not always result in reduced accuracy. The latter includes ResNet [10] and DenseNet [14], which minimize the inherent redundancy in dense model parameters and advance the development of a more lightweight network mode. Nevertheless, the aforementioned approaches mostly concentrate on reducing redundancy, whether in the channel or spatial dimensions, with the consequence that networks continue to encounter challenges associated with feature redundancy. SCConv appears to be a good solution to problems of this kind, as it exploits the redundancy of intermediate feature maps and reduces the parameters and computation without performance loss. SCConv uses a two-step procedure, spatial reconstruction unit (SRU) and channel reconstruction unit (CRU), to reduce the number of parameters and FLOPs (see Figure 4).

**Figure 5:**
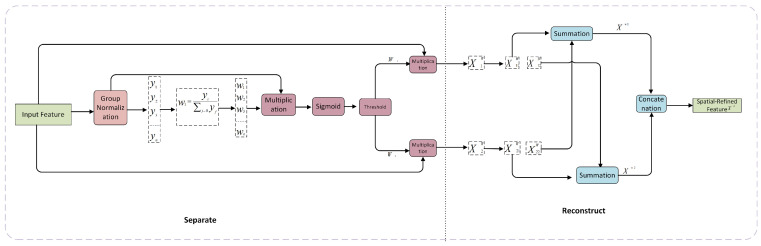
The architecture of the Spatial Reconstruction Unit (SRU).

Through these two procedures, the feature representation capability was also strengthened. The SRU can be considered a process of Separate-and-Reconstruct operation, as shown in Figure 5.

The objective of the separate operation is to distinguish and isolate relevant feature maps from less informative counterparts that correspond to the spatial content. The informative content of various feature maps was evaluated by using scaling factors in the group normalization (GN) layers [29]. The trainable parameters of the GN layers were used to quantify the spatial variation in the pixels within each batch and channel. The increased richness of spatial information is indicative of greater diversity in spatial pixels, which in turn contributes to the enlargement of the parameters. The weight values of the feature maps were subsequently adjusted and used to map the range between 0 and 1 by applying a sigmoid function. These values were then subjected to a gating threshold. Finally, the feature maps were given multiple informative and non-informative weights. The outcomes of the feature map times the noninformative weights were regarded as redundant. For the reconstruction operation, rather than directly including these two components, a cross-reconstruction process was used to effectively merge the weighted informative features and enhance the information exchange between them. Subsequently, the cross-reconstructed features were concatenated to obtain spatially refined feature maps.

**Figure 6:**
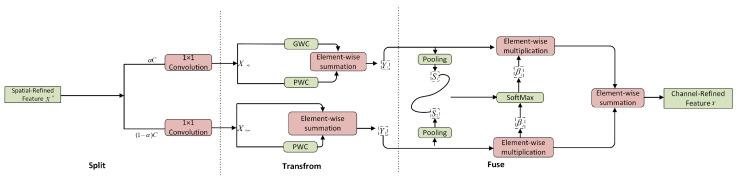
The architecture of the channel reconstruction unit (CRU).

Subsequently, the CRU method uses a split-transform-and-use approach (shown in Figure 6), and this was done to further reduce the duplication present in the spatially refined feature maps throughout the channel dimension. In addition, the CRU algorithm utilizes lightweight convolutional operations to extract valuable representative features, while efficiently eliminating redundant features by using inexpensive operations and feature-reuse techniques. Generally, CRU can be used alone or in conjunction with SRU operations. The proposed SCConv was established by organizing the SRU and CRU sequentially, resulting in a highly efficient convolution operation with the potential to replace ordinary convolutions.

### 3.4. Omin-Dimensional Dynamic Convolution for Multi-Dimensional Attention Mechanism

**Figure 7:**
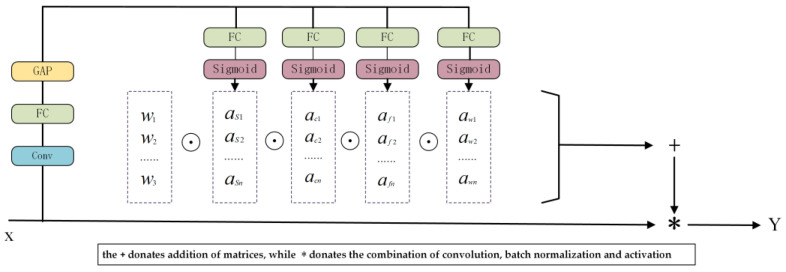
Schematic of an omni-dimensional dynamic convolution.

Traditional approaches to dynamic convolution often use attention techniques to dynamically adjust the weights of the convolution kernels in a single dimension of the kernel space. As shown in Figure 7, input variable x is subjected to an initial global average pooling (GAP) operation, and subsequently it undergoes further processing via fully connected (FC) layers and activation functions, namely, the Rectified Linear Unit (ReLU). Unlike traditional dynamic convolutions, ODConv has four separate header branches, which are each equipped with a fully connected layer and a Softmax or Sigmoid activation function. A mathematical representation of this process can be described by using (1): by integrating dynamic convolutions, the model acquires the capacity to flexibly modify the weights of the convolution kernels in response to the distinctive characteristics of the input data. This enhances the ability of the model to combine and integrate features. Consequently, the ability of the model to perceive and capture high-frequency information, such as intricate visual features and textures, was strengthened, resulting in improved performance and accuracy.

(1) $$y=(\alpha_{\omega 1}⨀\alpha_{f1}⨀\alpha_{c1}⨀\alpha_{s1}⨀W_{1}+\cdots +⨀\alpha_{\omega n}⨀\alpha_{fn}⨀\alpha_{cn}⨀\alpha_{sn}⨀W_{1n})\;*\;x.$$

The attention mechanism in the ODConv framework was designed to function across four dimensions of kernel space: location, channel, filter, and kernel. The use of complementary attention mechanisms in a convolution operation allows the integration of varying attention at each stage, resulting in diverse influences acting on each dimension of the input. This methodology enables more efficient use of geographical data and produces better results when collecting complex contextual information. The integration of ODConv into the slim-neck design successfully mitigated the negative consequences associated with the significant use of lightweight convolution kernels and depth-wise convolutions. This improved the capacity of the model to comprehend contextual information and spatial dimensions, which in turn improved the precision and resilience of job detection.

### 3.5. Structure of the Ship-Fire Net

**Figure 8:**
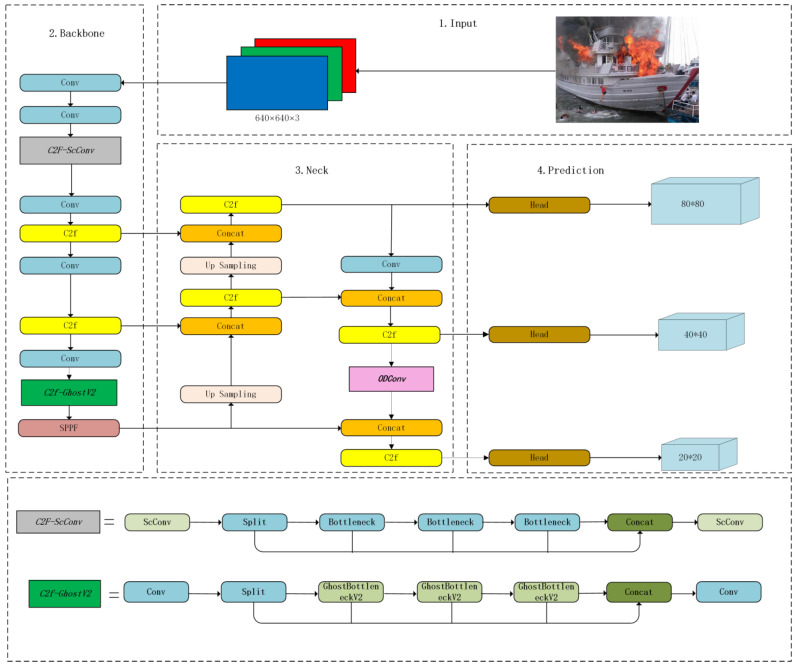
The architecture of proposed model (Ship-Fire Net).

Figure 8 shows the structure of the improved YOLOv8n (Ship-Fire Net) for ship fire and smoke detection. In the case of the backbone part, spatial and reconstruction convolution were used to replace the traditional convolution part in the first C2F module reduction of feature redundancy, which created a new C2F-SCConv module. The GhostNetV2 bottleneck was then used to replace the original bottleneck in the fourth C2F part, in order to achieve cheap operation with long-range attention. In the case of the neck part, omni-dimensional dynamic convolution was used for the multidimensional attention mechanism, which improved the precision and resilience of detection.

## 4. Data Collection and Experimental Setting

### 4.1. Ship Fire Data Set Collection

**Figure 9:**
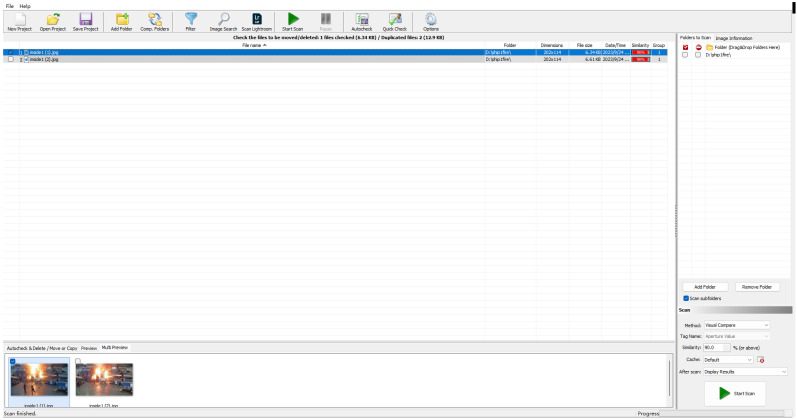
The pre-process before labeling using Visual Similarity Duplicate Image Finder.

Ship fires may occur inside ships, such as in cargo control rooms, fire control and storage rooms, or stevedore cabins. When these fires occur, the background is dissimilar to fires that occur on the deck. Therefore, to simulate fire in the vessels, we considered two types of datasets: fire inside vessels and fire outside vessels. When a fire occurs inside vessels, the first reaction of passengers or sailors is to put out the fire rather than record it with a camera, making the fire inside the ship slightly harder to find. The foundation of the dataset consists of two parts of the main job: searching the ship fire images and using labeling tools to locate the position of fire and smoke in the images. To achieve the first goal, large search engines like Baidu and Google are used to search keywords such as ‘ship + fire’, ‘ship + fire + rooms’, ‘ship + smoke’, and so on. Then, in order to save the time that would otherwise need to be committed to downloading the images one by one, python was used as the web crawler to download the images. All the images were checked for copyright for academic purposes. One problem in the collection of datasets is image repetition, as different websites may possess the same images, which may be downloaded by web crawlers. To improve the diversity of the dataset, all images are pre-processed before being labeled by using the Visual Similarity Duplicate Image Finder software (Figure 9). The version of it is 8.3.0.1. This software can change the score of similarity from 0–100 percent, and scan the whole folder to find and list similar images. Images with a similarity higher than 90 percent will be regarded as duplicates and will be deleted to ensure the quality of the datasets and performance of the algorithm.

**Figure 10:**
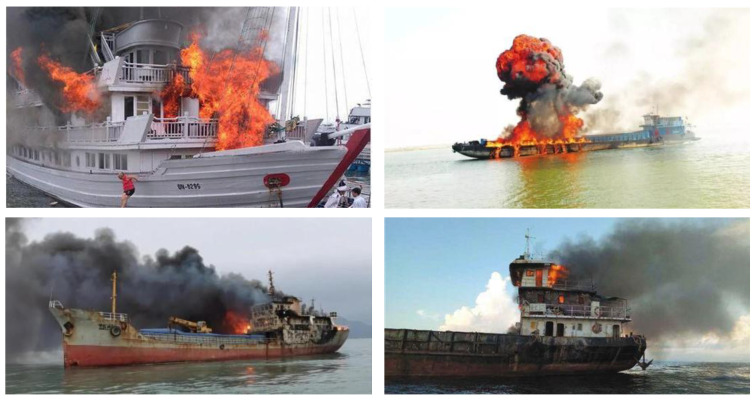
Example of the ship fire and smoke datasets (outside).

**Figure 11:**
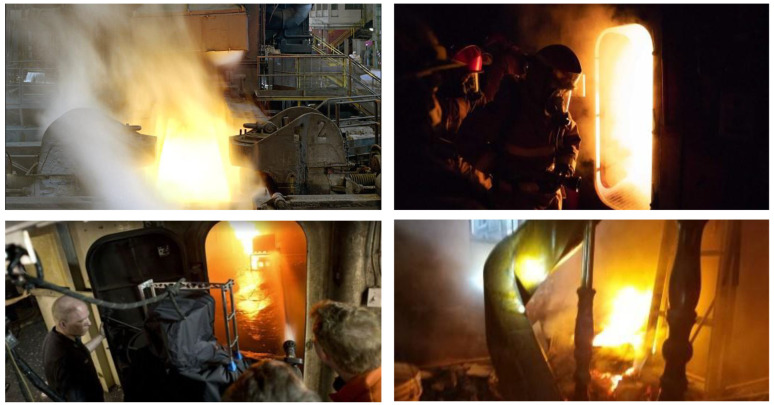
Example of the ship fire and smoke datasets (inside).

After these processes, 576 ‘inside’ and 3651 ‘outside’ fire images were selected. Samples of onboard fires are shown in Figure 10, and samples of fires inside rooms are shown in Figure 11.

After the collection of datasets, researchers then used ‘LabelMe’ to label fire and smoke in the images. All fires and smoke use a rectangular box to indicate their location and can then be extracted as feature maps for the CNN network. All labels were written in TXT format. The labels are made and located by researchers, and so the theme and content of images can be guaranteed to be ship fire. The labels and images were renamed as a one-to-one correspondence, and each image had its corresponding label. When compared to the other datasets of ship fire, our datasets are distinguished by the fact that they divide ship fire images into two categories: ship fire inside rooms and ship fire on board—this is done to ensure the algorithm can be used in the whole ship. In addition, the other datasets just consider the fire, while our dataset, in considering fire detection, considers both fire and smoke, which is important because smoke is an important signal of fire. All the datasets were uploaded to Google Drive, which is shown here: https://drive.google.com/drive/folders/118jiofV1EVOwY715T_zeCdk6zpkLErlp?usp=drive_link (accessed on 11 November 2023). There are two subfolders entitled ‘inside_’ and ‘outside_’, which respectively relate to onboard fire inside rooms and fire outside rooms. The images and labels are also numbered, and correspond to each other in ‘inside’ and ‘outside’ subfolders.

**Figure 12:**
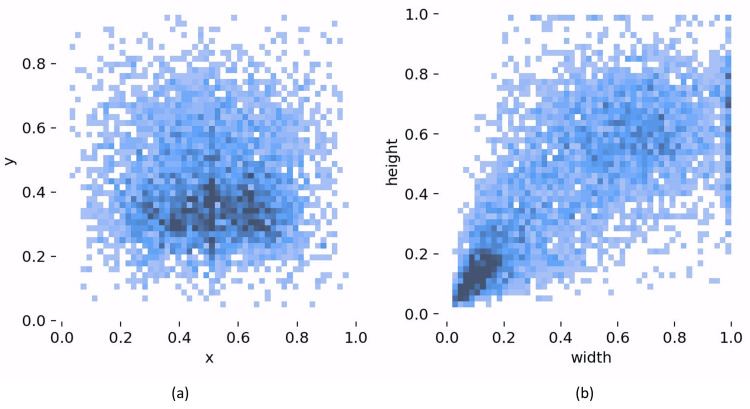
Visualization results of the analysis of the dataset. (**a**) Distribution of object centroid locations; (**b**) distribution of object sizes.

The images were folder images, and their corresponding labels were folder labels. They were divided as follows: 70% for training, 10% for testing, and 20% for validation, as shown in Table 2. The visualization results of the dataset analysis are presented in Figure 12. In Figure 12a, the distribution of the object centroid positions is depicted, with the abscissa and ordinate representing the centroid positions. Figure 12b illustrates the distribution of the object sizes, with the abscissa and ordinate representing the width and height of the object, respectively. The data revealed a higher concentration of targets in the middle area, and a majority of these targets were small. This observation aligns with real-world fire situations and substantiates the claim that our algorithm is effective in detecting fires during their early stages, when they have smaller dimensions.

### 4.2. Experimental Environment

The present study was carried out with an Ubuntu 20.04 operating system, employing an 11th Generation Intel i9-11950H central processing unit and an RTX A5000 graphics processing unit in Intel, Singapore. The GPU acceleration environment was established by using CUDA 11.3, and the network architecture was constructed by employing Python 3.9 and PyTorch 1.11.1. The study’s development platform was Visual Studio Code version 1.75.0.

### 4.3. Hyperparameter Setting

All experiments were performed by using the same listed hyperparameters to demonstrate the effectiveness of the proposed method, as shown in in Table 3.

### 4.4. Model Evaluation Metrics

The evaluation of model performance often involves the use of two criteria to assess its strengths and limitations. Recall, accuracy, and mean average precision (MAP) are typically used as measures for assessing a model’s detection capabilities. The metrics used to assess the detection speed and computational complexity of the model include parameters such as the floating point operations (FLOPS) and frames per second (FPS). In this study, the precision was used as a determinant to assess the accuracy of the model. Accuracy was defined as the ratio of correct positive predictions (TP) to the overall number of positive outcomes (TP + FP) projected by the model, and was significantly affected by the presence of a significant number of false positives (forest fire [26]). The range 0–1 was calculated with precision by using the following methodology:

(2) $$P(Precision)=\frac{TP}{TP+FP}.$$

Recall is defined as the proportion of correctly identified positive instances (true positives) relative to the sum of true positives and false negatives. The recall can be determined by using the following mathematical expression:

(3) $$R(Recall)=\frac{TP}{TP+FN}.$$

The F1-score is defined as the harmonic mean of Precision and Recall. The definition can be stated as follows:

(4) $$F_{1}=2\times \frac{Precison\times Recall}{Precison+Recall}.$$

The concept of average precision (AP) refers to a measure of precision that encompasses all elements within a specific category of pills, as outlined in Equation (5):

(5) $$AP=\int_{0}^{1}P\left(R\right)dR.$$

The MAP was calculated as the arithmetic mean of the average precision (AP) values obtained for each category in the dataset. This metric was used to assess the overall model performance. This definition is illustrated in Equation (6):

(6) $$mAP=\frac{1}{N}\sum_{i=1}^{N}AP_{i}$$

The complexity of each model was assessed by using characteristics such as FLOPs and FPS. The parameters of the CNN model refer to the variables obtained and modified by the network during training. The number of parameters in a CNN model is determined by its architectural configuration, which includes variables such as the number of layers, dimensions of filters, and number of neurons in each layer. Models with more layers and neurons often demonstrate a corresponding increase in the number of parameters. The total number of parameters in a CNN model is a crucial factor that significantly influences the complexity of the model, memory requirements, and training time. Models that are more complex and have a larger number of layers and neurons tend to have more parameters, and consequently require longer training times. In addition to these characteristics, the computational efficiency of the model is influenced by the number of floating-point operations per second. Floating-point operations are mathematical calculations that involve actual numbers with fractional components, and they play a crucial role in CNNs for various computational tasks that include convolutions, matrix multiplications, element-wise operations, and nonlinear activations. In the CNN context, standard procedures include the application of operations to the input data and the learnable parameters of the network, namely weights and biases. The results of these procedures include the generation of forecasts and the creation of characteristic representations. The use of floating-point operations is crucial for facilitating the effective processing and manipulation of numerical information inside CNNs, and these procedures enable precise and sophisticated calculations that are necessary for a range of activities, such as picture categorization, object recognition, and image synthesis. FLOPS pertains to the investigation of floating-point operations in CNNs within the wider context of scholarly inquiry, which involves scrutinizing the computing demands linked to the different layers and operations. This study provides an examination of optimization approaches to reduce computational complexity, and also gives insight into research into hardware accelerators and parallelization methodologies. A significant emphasis in this field of research is the development of algorithms and models that can effectively balance accuracy and computing efficiency. The acronym “FPS” refers to the measurement of the frequency at which frames, or visual representations, are processed or created inside a CNN model or computer vision system. The FPS measure is of great significance within the domain of CNNs because it plays a crucial role in assessing the computational efficiency and real-time capabilities of jobs related to image or video processing, which are both useful to assessments of the performance of the ship-fire networks.

## 5. Results and Discussion

### 5.1. Based Algorithms Selection

The training procedure for each experiment involved the utilization of six well-established object detection algorithms: Faster R-CNN ResNet50 [29], SSD MobileNet_V1 [30], SSD Inception_V2 [31], swin transformer [47], Densenet [48], YOLOv3-tiny [33], YOLOv5s [49], YOLOv7-tiny [50], and YOLOv8n [51]. These algorithms were specifically trained for fire detection by using internally generated datasets. The findings of these studies and their corresponding comparisons are summarized in Table 4.

YOLOv5s has superior recall compared to the other models; however, its accuracy, recall, F1, and map metrics are not as efficient as YOLOv8n. Both the precision and recall of yolov8n reached about 0.9 and the mAP@50 attained arrives 0.856. Taking into account the primary objective of this research, namely to develop a lighter and faster detection model, the speed of detection should be considered in conjunction with accuracy metrics. The weight of the model was then assessed by using the FLOPs and parameters. YOLOv8n’s parameters are only 3.01 million, the lowest among the algorithms. YOLOv8n can also detect 192 frames per second on average, which is faster than the others. While Inception_V2 may exhibit some benefits in terms of FLOPs, it falls far short of YOLOv8n in terms of parameters and FPS. In addition, YOLOv8n’s precision, recall and mAP@50 all exceed the Inception_V2 counterparts. The experimental findings for the five widely used detection algorithms indicate that YOLOv8n has notable benefits, in terms of both accuracy and efficiency. We therefore chose YOLOv8n as the initial model for future enhancements and improvements.

### 5.2. Ablation Tests Results

Eight ablation experiments were conducted to examine the impact of the various approaches. All experimental groups used identical datasets, training settings, and methodologies for training. The empirical findings are presented in Table 5.

The first row in Table 5 shows the results of the original YOLOv8n network without any improvement measures. Compared to the original YOLOV8n, all three new models became lighter and faster after the GhostNetV2, ODConv, or SCConv modules were inserted to replace the original part. Especially when GhostNetV2 was used in the algorithm, the FLOPS reduced to 7.85 (G) and FPS reached above 220. In addition, map@0.5, ODConv, and SCConv increased by 1.1% and 2.0%, respectively. After all three different improvements to the algorithm, a ship-fire net was formed. Compared to the original, it improves exponentially by approximately 3.4%, 3.1%, 3.2%, and 4.1% in terms of precision, recall, F1, and map@0.5%, respectively. In addition, the parameter flops were the lowest in the three models, while its FPS exceeded 280. In summary, after the improvements and modifications, the Ship-Fire Net’s precision and recall both reached about 0.93 and the detection speed also improved, as reflected by the decrease in the parameters and Flops and the increase of FPS.

**Figure 13:**
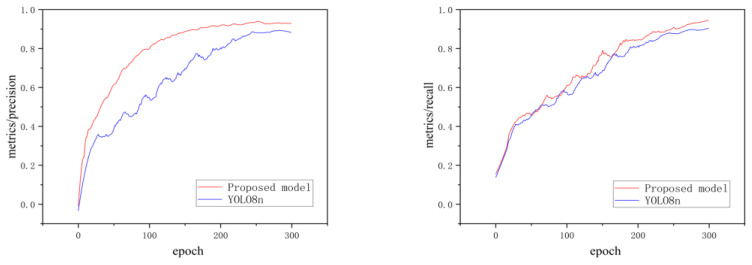
Precision–epoch and recall-epoch curve.

The precision–epoch and recall-epoch curves are shown in Figure 13. As the number of training epochs increases, both the precision and recall curves become larger for the two algorithms. When the number of epochs exceeds 200, the proposed model is significantly different from the original YOLOv8n model.

**Figure 14:**
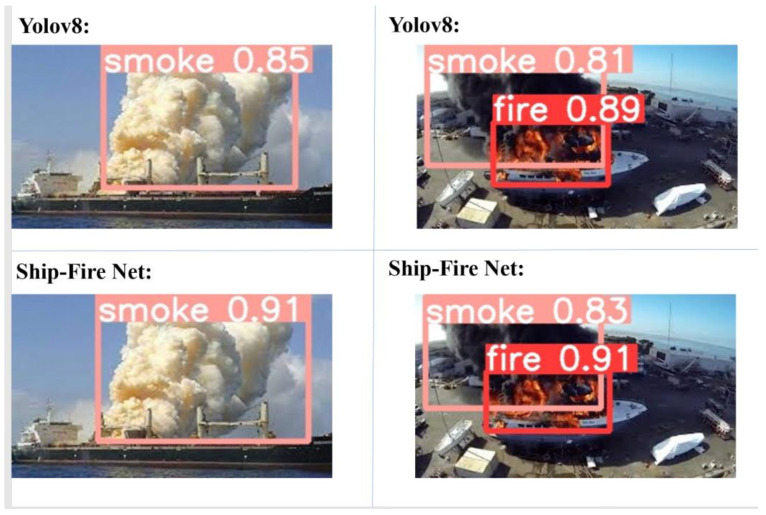
Results of Ship-Fire Net and YOLOv8n for outside images.

**Figure 15:**
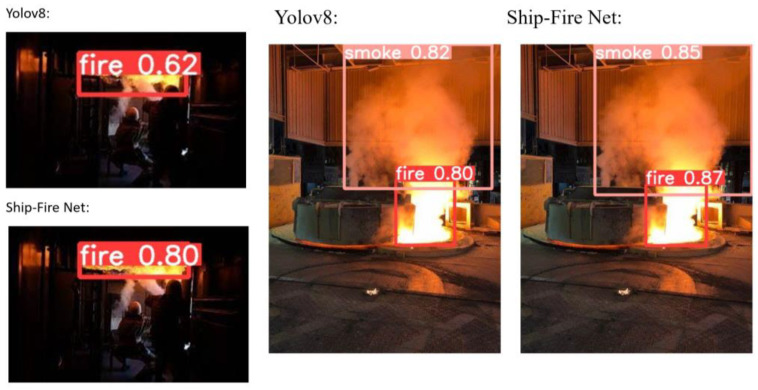
Results of Ship-Fire Net and YOLOv8n for inside images.

This system was tested in real-world scenarios with rooms inside the vessels and vessels. Figure 14 and Figure 15 illustrate fire detection under different conditions.

## 6. Discussions and Limitations

The success of the novel system can be attributed to the ability of the YOLOv8 model to localize fires and smoke, both in rooms and onboard. The efficiency and performance of a system may vary under various conditions, such as lighting variations, smoke, variable sensitive environmental factors, and obstructions. The foundation of the two categories of datasets and the labeling work of smoke and fire helped to achieve this, and ensure that the algorithm could be strongly implemented after the training process for fire and smoke detection purposes. When compared to other advanced object detection algorithms, YOLOv8 was found to provide higher-class results in detecting fires in bright and dark lighting, recognizing small fires and flames, and distinguishing between fire and non-fire scenarios. To make it lightweight, several improvements to GhostnetV2, SCConv, and ODConv were implemented. After these improvements, the parameters and FLOPs significantly improved, which also accounted for the higher FPS. We have also searched relevant studies about ship fire detection by using CNNs, and tried to use their algorithms for training on our datasets. Table 6 records recently published fire detection methods and compares them with the proposed method.

The results in Table 6 confirm that Ship-Fire Net showed great performance in both accuracy and detection speed. Compared to the Tiny-YOLOv2 model, YOLOv4-tiny model, YOLOv7 model, and YOLOv7-tiny model, the mAP@0.5 Of the Ship-Fire Net increased by 10.8%, 9.3%, 8.4% and 5.2%, respectively. Precision increased by 11.8%,6.8%,6.3%, and 5.9%, while recall enhanced by 13.9%, 10.2%, 8.6%, and 6.9%, respectively. In addition to flame detection, Ship-Fire Net also achieved a high detection accuracy rate for smoke, as shown by a mAP@0.5 that reached 0.884. The detection speed was also guaranteed to beabove 200 FPS, and our model is also the fastest one of the five. In conclusion, Ship-Fire Net outperforms the other algorithms that seek to achieve ship fire detection in mAP@0.5, accuracy, and recall, and also don’t manage to achieve lightweight real-time ship fire detection.

However, our dataset and algorithm still have some limitations. Firstly, our study only focused on the presence or absence of fire, without considering its extent and progression. This study is also confined to the realm of algorithms, and therefore neglects practical applications. During the course of the trial, several photos, including objects resembling fire, were correctly identified as fire. If a picture exhibits the presence of luminous sunshine, highly saturated yellowish-red lights, or incandescent bulbs such as fire, it may be identified as a depiction of fire. In addition, ships, and especially large cargo ships or large cruise ships, may have a large number of cameras to capture fire or smoke images from different angles for detection. However, on small ships the lack of cameras or detection systems may result in the algorithm not being applicable and useful. Last but not least, there is a significant category disproportion between the 576 interior ship fire images and the 3651 exterior ship fire images. This is because, when a ship fire happens, passengers or sailors who observe the fire will always try to first extinguish the fire rather than record it on phones or cameras. When a fire happens on board, passengers, whether on other ships or on land, may help to record the fire.

To deal with such limitations, we must not only focus on the occurrence of a fire but also conduct comprehensive investigations of post-fire circumstances, including by examining the progression of the fire and analyzing the crew’s evacuation routes. In addition, we intend to integrate the established techniques and algorithms to enhance the performance of the model. For instance, we may include long short-term memory (LSTM), a widely used approach in the domain of natural language processing, as well as the temporal difference method, which is often used in conventional moving object identification research. For fire-like objects, we will expand the datasets and create labels for fire-like objects to train the model, thereby reducing the false alarm rate. And in seeking t deal with the concern of long-distance fire or blocked fire in small ships, we will to combine our fire detection system with traditional temperature sensors and smoke sensors; with the help of traditional sensors, even long-distance fire or blocked fire can be detected in the early stage. And finally, in engaging the category disproportion problem, we will try to arrange drills about ship fire inside rooms and record them for further studies, as this will balance the category disproportion.

## 7. Conclusions

In conclusion, we propose an improved and faster version of a fire detection system for ships using the YOLOv8n architecture. Thorough experiments and system evaluation, we demonstrated that the proposed system is highly efficient in detecting real-time fires and smoke in challenging environments. To achieve this goal, we first attempted to create a dataset that contained both fire in the rooms of a ship and fire onboard, which ensures that the algorithm can be used and works well both onboard and in rooms. All images were checked for similarity and labeled manually. After comparing the performance of several selected advanced objections, we found that, although YOLOv5s had the best performance in precision, YOLOv8n had a better detection consequence for index recall, F1, and mAP@.5. In addition, YOLOv8n has the fastest detection speed of all advanced models, which enables it to achieve real-time ship fire detection. After improvements under the same settings, we trained and evaluated the ‘YOLOv8n + Ghostnetv2 + SCConv + ODConv’ (Ship-Fire Net) and other competitors in ablation tests. The results indicate that the proposed algorithm exhibits the best performance for accuracy and speed. The accuracy and recall of our proposed Ship-Fire net are 0.931 and 0.938 respectively, and its F1 score is 0. 934. When compared to the original YOLOV8n, the modified algorithm was found to outperform it in terms of precision, recall, F1, and exponentially, by 3.4%, 3.1%, and 3.25% respectively, which underlined it would be able to detect and locate fire and smoke more accurately. In addition, the proposed model had lower FLOPs and parameters, and its FPS reached 280, which satisfied the requirements for real-time ship fire detection. After comparison with the other algorithms proposed for ships, our algorithm was found to have a wide usage range, the highest FPS, and to be the lightest. Finally, our algorithm can detect both fire and smoke, and its mAP@50 reaches 0.911 and 0.884 respectively, which proves that our model can effectively detect fire and its derivative(smoke). The detection of smoke can facilitate the detection of flame and improve the reliability of the detection system.

## Figures and Tables

**Table 1:** Summary of research that used CNN for ship fire detection.

Name	Based Algorithm	Datasets	Advantages	Disadvantages	FPS
Park et al. [19]	YOLOv2-tiny	5259 images	High accuracy	Only detects fire; smoke is ignored; it focuses on the fire in the engine room.	117
Wu et al. [21]	YOLOv4-tiny	2160 images	Use land dataset to train the model and transfer the weights	Small datasets for ship fire; data augmentation is used to enlarge datasets.	138
Avazov et al. [22]	YOLOv7	4622 images	Both fire inside and outside vessels are used	Data augmentation is used to enlarge datasets; some data are video frames and may possess high similarity.	175
Zhu et al. [45]	YOLOv7-tiny	12,986 images	Large datasets	The fire is simulated; the algorithm ignores the fire in other rooms and the deck.	189
Ours	YOLOv8n	4227 images	Both fire and smoke are detected. All images are checked for similarity. Fast detection speed.	Inside and outside fire images for ships show quantity variance	286

**Table 2:** Distribution of fire images in the dataset.

Dataset	Training Images	Testing Images	Validation Images	Total
Fire in ship room	2556	730	365	3651
Fire on board	403	115	58	576

**Table 3:** Training parameters of the ship fire detection model.

Training Parameters	Details
Epochs	300
batch-size	16
image-size (pixels)	640 × 640
Initial learning rate	0.01
Optimization algorithm	SGD
Pre-training weights	None

**Table 4:** Results of classical advanced deep learning algorithms.

Models	Precision	Recall	F1	mAP@50	Parameters (m)	FLOPS (G)	FPS
ResNet50	0.524	0.531	0.53	0.494	42.25	430.9	30
MbileNet_V1	0.675	0.542	0.60	0.581	5.53	13.2	72
Inception_V2	0.689	0.701	0.69	0.644	13.32	7.6	53
swin transformer	0.741	0.681	0.71	0.623	28	47.1	45
densenet	0.671	0.612	0.64	0.563	66.76	32.7	35
YOLOv3-Tiny	0.797	0.821	0.81	0.745	8.7	12.9	140
YOLOv5s	0.901	0.855	0.88	0.814	7.02	16	114
YOLOv7-tiny	0.857	0.7921	0.82	0.758	6.02	13.2	143
YOLOv8n	0.897	0.907	0.90	0.856	3.01	8.2	192

**Table 5:** Results of ablation experiments.

Models	Precision	Recall	F1	mAP@0.5	Parameters (m)	FLOPS (G)	FPS
YOLOv8n	0.897	0.907	0.902	0.856	3.01	8.2	192
YOLOv8n + ODConv	0.913	0.912	0.912	0.867	3	8.1	213
YOLOv8n + GhostNetV2	0.908	0.916	0.912	0.859	2.72	7.85	222
YOLOv8n + SCConv	0.915	0.914	0.914	0.876	3	8.1	217
YOLOv8n + GhostNetV2 + ODConv	0.926	0.929	0.927	0.862	2.71	7.75	263
YOLOv8n + GhostNetV2 + SCConv	0.924	0.932	0.928	0.887	2.71	7.75	278
YOLOv8n + ODCONV + SCConv	0.928	0.933	0.930	0.841	2.99	8	270
YOLOv8n + GhostNetV2 + ODConc + SCConv (Ship-Fire Net)	0.931	0.938	0.934	0.897	2.69	7.65	286

**Table 6:** Comparison of detection consequence of the ship fire detection algorithms proposed by others and Ship-Fire Net.

Name	Based Algorithm	FPS	Precision	Recall	F1	mAP@0.5
Park et al. [19]	Tiny-YOLOv2	117	0.813	0.799	0.806	0.789
Wu et al. [21]	YOLOv4-tiny	138	0.863	0.836	0.849	0.804
Avazov et al. [22]	YOLOv7	175	0.868	0.852	0.860	0.813
Zhu et al. [45]	YOLOv7-tiny	189	0.872	0.869	0.870	0.845
Ship-Fire Net	YOLOv8n	286	0.931	0.938	0.934	0.897 (fire at 0.911 while smoke at 0.884)

## Data Availability

Data are contained within the article.
